# Mind wandering via mental contrasting as a tool for behavior change

**DOI:** 10.3389/fpsyg.2013.00562

**Published:** 2013-09-02

**Authors:** Gabriele Oettingen, Bettina Schwörer

**Affiliations:** ^1^Psychology Department, New York UniversityNew York, NY, USA; ^2^Department of Psychology, University of HamburgHamburg, Germany

**Keywords:** mind wandering, thinking about the future, fantasies, mental contrasting, behavior change intervention, self-regulation, motivation

## Abstract

When people engage in mind wandering they drift away from a task toward their inner thoughts and feelings. These thoughts often circle around people's personal futures. One assumed function of mind wandering is that it aids problem solving and planning for the future. We will discuss different forms of mind wandering and their effects on problem solving and behavior change. While solely fantasizing about a desired future leads to poor problem solving and little behavior change, mind wandering in the form of mental contrasting leads to skilled problem solving and substantial behavior change. In mental contrasting, people first envision the desired future and then imagine the obstacles that need to be surmounted to reach said future. Mental contrasting instigates behavior change by modulating the strength of associations between future and reality and between reality and instrumental action. Intervention research shows that mental contrasting can be taught as a cost- and time-effective self-regulation strategy of behavior change. The findings have implications for research on mind wandering, problem solving, and on creating effective interventions of behavior change.

Mind wandering (Singer, 1966; Antrobus, 1968; Klinger, 1990; Giambra, 1995; Teasdale et al., 1995; Wegner, 1997; Smallwood and Schooler, 2006) occurs when the attention shifts away from a current task or the present situation to one's inner thoughts and feelings. For example, people may read a book without absorbing the book's content by thinking about something unrelated to the book's story. Mostly, these free thoughts are not triggered by stimuli within the environment but by stimuli within the person herself. Unfulfilled goals or current concerns are assumed to be one important source of mind wandering (e.g., Singer, 1966; Klinger, 1975, 1990; Smallwood, 2010). Studies investigating the contents of mind wandering support this assumption: People engaging in mind wandering oftentimes think about their current concerns and their free thoughts tend to be future oriented (e.g., Berntsen and Jacobsen, 2008; Smallwood et al., 2009a; Baird et al., 2011; D'Argembeau et al., 2011). One hypothesis implies that mind wandering about personal concerns and future states supports future planning and the resolving of everyday life problems (Klinger, 2009; Schooler et al., 2011). So far, research in the field of mind wandering has not directly approached this hypothesis, and as Smallwood and O'Connor (2011) stated, future studies answering the question of how the content of mind wandering affects individuals' functioning, are needed. In order to shed light on the function of mind wandering, we will describe the results of over 15 years of research about free thoughts and images about the future, and how they impact individuals' cognition, emotion, and action.

## Positive fantasies

The self-help literature clearly advises us to think positively in order to become a happy and successful person. Studies on fantasies about the future (i.e., free thoughts and images; Oettingen and Mayer, 2002; Oettingen, 2012) and their effects on effort and success, however, speak differently: The more positive a person's fantasies about the future, the less successful she is in realizing these fantasies. This pattern of results appears in the health, interpersonal, and academic domain, across ages and cultures, and for different measures of fantasies as well as for different indicators of effort and success (for a review, see Oettingen, 2012). For example, the more positively overweight women fantasized about losing weight, the fewer pounds they lost over the course of one year (Oettingen and Wadden, 1991), and the more positively patients undergoing hip replacement surgery fantasized about their recovery, the less well the physical therapists rated their actual recovery 2 weeks later (Oettingen and Mayer, 2002). Positivity of fantasies predicted low success in entering a romantic relationship, low course grades in an upcoming exam, and over the course of 2 years a poor transition into work life (Oettingen and Mayer, 2002; H. B. Kappes et al., 2012). Compared to relevant control groups, induced positive fantasies led to lower mastery of everyday life (H. B. Kappes and Oettingen, 2011) and less willingness to contribute to charity, whether giving was measured by donating money, effort, or time (H. B. Kappes et al., 2013).

### Positive fantasies: mechanisms

Positive fantasies about a wished for future allows a person to enjoy future success already in the here and now, and thus it appears that no effort for fulfilling the wished for future is needed. Indeed, in four studies, inducing positive fantasies led to lower energization and lower effort than questioning fantasies, negative fantasies, or factual thoughts (H. B. Kappes and Oettingen, 2011). In these studies, energization was measured by subjective feelings as well as by objective measures (e.g., systolic blood pressure). The results imply that when aiming to realize a desired future one should not let one's mind solely wander to a positive future; rather one should integrate in one's fantasies a clear sense of reality. We present mental contrasting of future and reality, a self-regulation strategy of behavior change that uses positive future fantasies as the background against which obstacles of current reality are imagined: The strategy produces behavior change as people now effortfully try to overcome the obstacles to reach the wished for future.

## Mental contrasting

Mental contrasting (Oettingen, 2000, 2012; Oettingen et al., 2001) is a problem solving strategy that leads to selective behavior change. In mental contrasting individuals first positively fantasize about a wished for future (e.g., solve an ongoing interpersonal conflict) and then mentally elaborate the current reality that stands in the way of realizing the envisioned future (e.g., one's shyness). By imagining the future and thereafter imagining obstacles of reality, the future links to the reality, revealing that in order to realize the wished for future, one has to act on the current reality (e.g., to solve the interpersonal conflict one needs to overcome present shyness). As a consequence, one's expectations of being able to overcome the obstacle to attain the desired future will determine one's behavior: high expectations lead to increased effort and success and low expectations lead to decreased effort and success; the latter causing people to free up resources by letting go of pursuing unrealistic wishes.

Fantasy Realization Theory (Oettingen, 2000, 2012) specifies two additional modes of thought: solely thinking about the positive future (indulging) or solely thinking about the obstacles of current reality (dwelling). Indulging in the desired future ignores possible obstacles and therefore conceals the necessity to act. Dwelling on present reality, on the other hand, does not give direction of where to go. Behavior therefore stays unchanged and independent of a person's expectations of success.

An ample amount of studies show the different effects of mental contrasting vs. indulging and dwelling on behavior change. For example, college students named their most important interpersonal concern and indicated their expectations of resolving the concern. They were then randomly assigned to one of three conditions: mental contrasting, indulging, or dwelling. When measuring their feelings of energization right after the experiment, and then 2 weeks later, participants with high expectations increased in energization and acted immediately, those with low expectations decreased in energization and delayed their action. Students in the indulging and dwelling conditions did not change, neither in their energization nor in their immediacy of action, regardless of whether their expectations were high or low (Oettingen et al., 2001). In other words, indulging and dwelling led individuals to invest too little when chances of success were high and to invest too much when chances were low. This pattern of results has been replicated in different life domains (e.g., interpersonal, health, academic), for short term as well as for long term goals (e.g., giving a speech, female doctoral students combining career and childrearing), for people of different ages and cultures (e.g., Germany and the US), and for different measures of goal pursuit (e.g., cognitive, emotional, and behavioral; for an overview, see Oettingen, 2012).

### Mental contrasting: spontaneous use

Studies (H. B. Kappes et al., 2011; Sevincer and Oettingen, 2013) investigating which mode of thought participants spontaneously engage in, found that individuals rather indulge than mental contrast. These findings come as no surprise because mental contrasting is cognitively more demanding than other modes of thought (Achtziger et al., 2009). In contrast to indulging and dwelling, it involves both images about the desired future and images about the present reality. Like mental contrasting, mind wandering also involves thoughts about both the future and the present (e.g., Baird et al., 2011; Stawarczyk et al., 2011; Song and Wang, 2012). Thus, people should mind wander by indulging and dwelling, but also by mental contrasting. Which factors then influence whether mind wandering comes in the form of indulging or dwelling vs. mental contrasting?

Contextual factors influence the degree to which people spontaneously mental contrast. Specifically, in six studies H. B. Kappes et al. (2011) investigated the effects of mood on spontaneous mental contrasting. Assessing modes of thought in participants' writings about a personal concern, the authors found that sad mood as opposed to happy mood and neutral mood fostered mental contrasting. It is important to note that spontaneous mental contrasting, just like experimentally manipulated mental contrasting, led to expectancy-dependent goal commitment (H. B. Kappes et al., 2011; Sevincer and Oettingen, 2013).

When measuring spontaneous mental contrasting, we asked participants to mentally elaborate on their wishes and concerns, but did not catch them while mind wandering. However, as the frequency of mind wandering is associated with being unhappy (e.g., Kane et al., 2007; Smallwood et al., 2009b; Killingsworth and Gilbert, 2010; Mar et al., 2012), the question arises of whether unhappy mood triggers mind wandering in the form of mental contrasting. If so, we would speculate that mind wandering when triggered by contextual factors such as sad or unhappy mood may work as a vehicle for problem solving and behavior change. Other contextual factors than mood such as prior experience or habitual use with mental contrasting may also influence the frequency of mental contrasting when mind wandering. Specifically, if a person is skilled in mental contrasting or habitually engages in it, it should be less cognitively demanding and thus may occur more likely during mind wandering. Similarly, a high working memory capacity should allow a person to mental contrast during mind wandering.

### Mental contrasting: mechanisms

To explain the effects of mental contrasting on behavior change, research has identified three sets of mechanisms: cognitive changes, motivational changes, and people's response to set-backs. Regarding cognitive mechanisms, mental contrasting changes (a) the strength of the association between future and reality (b) the strength of the association between reality and the actions to overcome the reality and (c) the meaning of reality. Importantly, all these changes mediated the effects of mental contrasting on behavior change. Firstly, mental contrasting (vs. relevant control conditions) strengthens the association between future and reality when expectations are high, and weakens it, when expectations are low (Oettingen, 2012). Secondly, mental contrasting also strengthens the association between reality and instrumental means to overcome the reality. For example, for a student who expects a high course grade in an important upcoming exam the obstacle of reality (e.g., being invited to a party) is closely associated with the action to overcome the obstacle (e.g., stay home and study; A. Kappes et al., 2012a). Thirdly, mental contrasting changes the meaning of reality: Only when expectations are high is the reality interpreted as an obstacle (e.g., the party on Saturday is not a fun event, but an obstacle to obtaining the high grade). Mental contrasting even helps to detect obstacles that stand in the way of fulfilling one's wishes. For example, young chess players with high expectancies who were induced to mental contrast detected a chess piece more readily as an obstacle to winning a chess match than did respective chess players in the control condition (A. Kappes et al., 2013).

The motivational mechanism bridging the effects of mental contrasting and behavior change is energization (measured by subjective feelings as well as by objective measures like systolic blood pressure): When expectations are high, people feel more energized and systolic blood pressure increases, when they are low, people feel less energized and blood pressure decreases. Other modes of thought produce no change. Importantly, changes in energization mediate the effects of mental contrasting on behavior change (Oettingen et al., 2009).

A third mechanism that renders mental contrasting effective is how people respond to set-backs. A. Kappes et al. (2012b) tested whether mental contrasting affects responses to goal-relevant negative feedback. Mental contrasting promoted the processing of useful information entailed in negative feedback which in turn helped participants to form plans for implementing behavior change. Mental contrasting also protected the self-view of competence against negative feedback and facilitated optimistic attributions for negative feedback. These results suggest that mental contrasting regulates effective responses to negative feedback to initiate and sustain behavior in line with expectations of success.

In sum, mental contrasting changes non-conscious cognition, energization, and responses to negative feedback. The exercise of mental contrasting itself, however, is a conscious procedure that is cognitively demanding. Indeed, Achtziger et al. (2009) using MEG tested mental contrasting vs. indulging vs. resting in a within design showing that mental contrasting is a cognitively demanding strategy that goes along with heightened mental activity exactly in those areas that are suggested by Fantasy Realization Theory: In the areas associated with holistic thinking and intention formation, working memory and episodic memory as well as with intense visualization. In contrast, indulging showed to be a non-demanding, undirected way of thinking about the future similar to resting (Achtziger et al., 2009).

### Mental contrasting: interventions

Based on the effects and mechanisms of mental contrasting discovered in the laboratory, we created intervention studies testing whether the strategy can be successfully used in the field. These studies show that mental contrasting can be easily taught and that people can apply the strategy by themselves. People can use mental contrasting regarding wishes of any content and pertaining to any life domain (e.g., academic, interpersonal, health). People can use mental contrasting across their life span and regardless of which socio economic status and cultural background they belong to. Mental contrasting helps to resolve short-term concerns and fulfill long-term wishes as it provides clarity and direction of what one wants to achieve and what is necessary to let go of. For example, mental contrasting led health care professionals to successfully manage their everyday life (Oettingen et al., 2010), disadvantaged elementary- and middle-school children to increase their school performance (Gollwitzer et al., 2011), patients suffering from type 2 diabetes to improve their self-care (Adriaanse et al., 2013), and middle aged, overweight fishermen of low socio-economic status to engage in more physical activity (Sheeran et al., 2013). In a standard dyadic negotiation game, mental contrasting participants found more integrative solutions than indulging, dwelling, and control participants, and were fairer to their partners (Kirk et al., 2011). Importantly, using mental contrasting in one domain helped behavior change in other domains. Students who were induced to mental contrast regarding eating fewer calories over the course of 2 weeks ate not only fewer calories but also increased their physical activity (Johannessen et al., 2012).

### Mental contrasting with implementation intentions

As described before, mental contrasting strengthens the association between the reality and the action to overcome the reality (A. Kappes et al., 2012a). But even though mental contrasting strengthens the association between reality and instrumental action as well as subsequent behavior change, people may stumble on the way to reaching the wished for future, especially when this future entails changing an engrained behavior (Webb and Sheeran, 2006; Adriaanse et al., 2010). Implementation intentions should prevent such stumbling. Therefore, we combined mental contrasting with implementation intentions (MCII), reasoning that mental contrasting effects on behavior should be even more evident when people in addition explicitly form implementation intentions. In short, we created Mental Contrasting with Implementation Intentions (MCII), a strategy encompassing both, mental contrasting and forming implementation intentions.

Implementation intentions (Gollwitzer, 1993, 1999) are plans that spur goal pursuit by linking a specific situation with a goal-directed action. They come in the form of “if situation x, then I will perform behavior y.” The effects of forming implementation intentions have been supported in a great number of studies across life domains (for reviews, see Gollwitzer, 1999; Gollwitzer and Sheeran, 2006). However, implementation intentions require determined goal pursuit, clarity of the critical situation (if-part) and clarity of the goal-directed action (then-part). Mental contrasting meets exactly these requirements. It creates determined goal pursuit, reveals the critical situation (obstacle of reality), and links the reality with the instrumental action to overcome the reality (goal-directed action). Applying mental contrasting with implementation intentions (MCII) indeed led to more behavior change than either strategy alone (Adriaanse et al., 2010; Kirk et al., 2013). For example, MCII led to improvement in attendance and course grades of children from disadvantaged backgrounds (Duckworth et al., 2013), in the homework of children at risk for ADHD (Gawrilow et al., 2013), and in the self-discipline of adolescents preparing for standardized tests (Duckworth et al., 2011). It helped to reduce insecurity-based behaviors (e.g., looking through the partner's phone log) and increased commitment in romantic relationships (Houssais et al., 2013). It also led to more physical exercise over a time period of 4 months and to a healthier diet over the period of 2 years (Stadler et al., 2009, 2010). Finally, mental contrasting helped chronic pain patients to become more physically active during rehabilitation and 3 months thereafter (Christiansen et al., 2010).

## Conclusion

In this review we shed light on the question of how different modes of self-regulatory thought affect behavior change. We first reported research about free thoughts and images and their impact on effort and success. Specifically, the more positive people's free thoughts and images about their desired futures, the more they failed to exert the necessary effort to attain these futures and the less successful they were—over the course of few weeks to several years. We then presented the strategy of mental contrasting that uses free thoughts and images to achieve prudent (expectancy-dependent) behavior change, regardless of whether the strategy is induced or used spontaneously. Mental contrasting, a conscious, and cognitively demanding strategy develops its effects on behavior change through non-conscious cognitive and motivational mechanisms, as well as by equipping people to effectively respond to set-backs.

Recent intervention studies show that mental contrasting can be easily taught and applied; it can be used by people of all ages and backgrounds to master their every-day life and fulfill their long-term wishes. Mental contrasting is particularly effective in providing sustained behavior change when combined with forming implementation intentions. The self-regulation strategy of mental contrasting adds to the traditional methods of behavior change. The latter have focused on changing people's beliefs as well as the content and structure of their goals (for a review, see Bargh et al., 2010). Mental contrasting, in contrast, focuses on the stream of thought people generate in response to identifying the outcome of their dearest wishes and the obstacles that hold them back from fulfilling these wishes. Importantly, the findings underpin the assumption that the content of mind wandering is worth examining in order to better understand how behavior change comes about.

### Conflict of interest statement

The authors declare that the research was conducted in the absence of any commercial or financial relationships that could be construed as a potential conflict of interest.
